# Increased Yield Surplus of Vetch-Wheat Rotations under Drought in a Mediterranean Environment

**DOI:** 10.1100/2012/658518

**Published:** 2012-05-02

**Authors:** Panagiotis Dalias

**Affiliations:** Agricultural Research Institute, P.O. Box 22016, 1516 Nicosia, Cyprus

## Abstract

This paper presents results of a plot-scale field experiment aiming at the comparative evaluation of agricultural practices and agricultural systems as far as their performance in very-low-rainfall conditions is concerned. Wheat was seeded after common vetch, treated in three different ways, after fallow or after the incorporation of dried sewage sludge or straw. Grain and straw yields and grain characteristics were always compared with conventional wheat monoculture without any additional organic inputs. Results showed a clear positive effect of vetch on next year's wheat yield and an increase in grain protein. Not only did the exceptionally dry season mask this effect, but also vetch-wheat systems were proved to be more effective in restraining wheat yield reductions, which are unavoidable under drought, marking these systems the most promising for improving sustainability and stability of rainfed agriculture.

## 1. Introduction

Cereals continue to be the most extensive type of cultivation in most Mediterranean countries. Although not very profitable, they are important for local consumption, and most significantly they provide roughage to the developed animal industry. As they are rarely under irrigation, their yields rely primarily on rainfall, which is the main limiting factor. Drought stress during the growing season and eventually available water stored in soil are the main factors explaining yield fluctuations in these countries [1, 2].

Agronomic stability and sustainability considerations keep vigorous discussions on best agricultural practices and cropping system suitability. These discussions exceed productivity concerns, including a number of environmental problems such as the decline of biodiversity, eutrophication of surface waters, increase in nitrate concentration of the underground water, and erosion [3].

Breaking cereal monoculture by incorporating legumes in rotation systems has long been proven, in many parts of the world, to have fertility and environmental advantages. Increase in sustainability and yield arising from the adoption of cereal-legume rotation has extensively been studied also in the dry regions of the Mediterranean basin [4]. The main conclusions of these studies indicate the importance of legume in increasing soil N availability for the subsequent cereal due to the transfer of biologically fixed N, to the N-sparing effects of the legume, and to less immobilization of nitrate during the decomposition of legume residues [5]. Moreover, legumes, such as lentils and vetch, were shown to not deplete soil moisture to the same extent as a cereal crop, leaving some residual soil moisture for the succeeding crop in the rotation [1]. Legumes also contribute to pest and disease control of subsequent cereals in rotations [6].

Better water economy and improved N nutrition together with the maintenance of soil organic matter are also objectives aimed at by producers using fallow or sewage sludge and straw residue amendments [7–9]. The decision for a farmer to improve the physical, chemical, or biological properties of the soil is greatly influenced by the necessary know-how and by technoeconomic analysis, which includes local availability of organic materials as well as transportation and handling costs.

Nevertheless, the performance of these cropping systems under severe crop season droughts has not sufficiently been studied. Droughts, which can be termed either as shortening of the rain season or as decrease of total rainfall, are going to be more frequent according to most climate change predictions [10], so the stability in yields offered by these systems in such conditions would be crucial for their eventual acceptance by farmers. It is underlined that even if other priorities, especially environmental ones, may ultimately influence agricultural management decisions, productivity and stability considerations inevitably play a key role as growers give priority to their annual returns.

The present study aims at the comparative evaluation of agricultural practices and agricultural systems as far as their performance in very-low-rainfall conditions is concerned. It focuses, in particular, on the comparison, under these conditions of wheat production potential and grain quality characteristics arising from the incorporation of straw or sewage sludge in soil or from the inclusion of wheat in rotation systems containing one year of vetch cultivation or fallow. The treatments compared are generally considered as having a positive effect on soil quality and productivity [11, 12] and are the ones that, to a smaller or greater extent, are already being applied in dryland agriculture of Mediterranean countries.

## 2. Materials and Methods

The experiment was carried out in an experimental station of the Agricultural Research Institute (ARI) of Cyprus near the city of Paphos. Soil in the study site is characterized as clay loam (Vertic Luvisol), with CaCO_3_ content of 15%, pH of 7.9, and organic matter content of 0.9% (Table 1).

The experiment was a two-factorial split-plot with four replications (randomized block design). The main plots were the different agricultural practices or agricultural systems, and the subplots were two tillage treatments, in a randomized design. The subplot size was 2.5 m × 15 m = 37.5 m^2^ and the main plot size was 5 × 15 m^2^ = 75 m^2^ extended in a total area of about 0.5 ha. The subplots were strips of land cultivated with two different tillage machines.

 The main treatment characteristics are given below together with the abbreviations with which they are quoted in the rest of the text. In three out of seven treatments, plots were seeded with durum wheat (*Triticum turgidum durum* var. Hekabe, purified variety enlisted in the national catalogue of varieties of Cyprus) during two consecutive years (continuous wheat cultivations), another three were seeded with vetch (*Vicia sativa* L. var. Kimon, variety enlisted in the national catalogue) the first year followed by wheat the next year, and in the last one, plots were left fallow the first year followed by wheat.

The same management practices, rotation systems, and experimental design were applied in two adjacent fields of the same soil type, with one of them starting two years after the first. Hence, test crop yields, that is, wheat yields, at the second year of each cycle, were obtained in 2008 in one field and in 2010 at the other. However, results from 2010 refer only to grain yields and will be used simply as an indication of performance of agricultural systems under normal-rainfall conditions.

**Figure 1:**
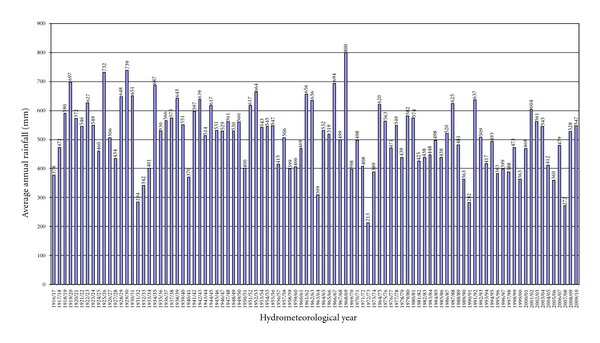
Time series of the annual area average precipitation in Cyprus (1917–2010).

The average annual rainfall in 2007/08 in Cyprus was 272.3 mm or 54% of the normal, which is estimated by the World Meteorological Organization for the years 1961–1990. This rainfall was the second lowest since the beginning of the previous century (Figure 1). Monthly rainfall during all months of the growing season was well below normal, and in most other districts of Cyprus apart from Paphos, where the experiment took place, it was not sufficient to support production of cereals. Meteorological data for the study field, as they were taken from the closest weather station, showed generally a smaller local normal annual rainfall (430 mm) than the average of the island but fluctuations over the years are largely well represented by those shown in Figure 1.

The seven treatments of the experiment were as follows:

1. Conventional treatment (abv. “cnvl”): plots were fertilized at a rate of 300 kg ha^−1^ year^−1^ of a 20-20-0 fertilizer as basic fertilization and 90 kg ha^−1^ year^−1^ of a 34.5-0-0 fertilizer as top dressing.
2. Straw incorporation treatment (abv. “strw”) straw in Cyprus is most frequently baled after harvesting, removed from the field and used as forage. In this treatment straw was incorporated in soil at a rate of 5.3 t ha^−1^ for the first year. For the second year, the amount of straw added was that harvested in the plots of this treatment, after its temporary removal for weighing. Plots were also added also inorganic fertilizers as in conventional treatment.
3. Vetch for hay treatment (abv. “vhay”): plots were seeded with vetch the first year, received only for that year 100 kg ha^−1^ of a phosphorus 0-48-0 fertilizer, and were left without inorganic fertilization for the following year of wheat crop. Above-ground biomass of vetch was cut during early stages of flowering and removed from experimental plots.
4. Vetch incorporation treatment (abv. “vinc”): plots were seeded with vetch the first year and treated as previously mentioned with no nitrogen fertilization, but above-ground biomass was incorporated in soil during flowering using rotavator.
5. Vetch for seed treatment (abv. “vgrn”): plots were seeded with vetch the first year and treated as in treatment 1, but vetch was-left to seed before harvesting and removal of above-ground biomass.
6. Fallow treatment (abv. “falw”): plots were left fallow during first year followed by wheat the second year. Wild vegetation was mechanically destroyed at the beginning and the middle of the growing season. Wheat was fertilized as in “cnvl” and “strw.”
7. Sewage sludge application treatment (abv. “sldg”): sludge, some characteristics of which are shown in Table 1, had been air-dried in the municipal waste water treatment plant for more than six months. It was added to soil only the first year at a rate of 15 t ha^−1^ (dry weight basis). Plots which received sludge were not given any other fertilizer during the whole period of the experiment.

Seeding was carried out at the end of November or beginning of December depending on soil moisture conditions. Soil samples properties shown in Table 1 were taken one week before first year's seeding. After harvesting, grain and straw yield was estimated and their contents in N were measured (Kjeldahl N). This allowed estimates of grain protein content (%N × 5.7).

Results and discussion below refer only to the comparison between agricultural practices and systems (main plots in the experimental design) and not to the effect of tillage (subplots), which is given no further analysis. However, this analysis showed that there was no interaction between the two tillage systems in relation to differences between treatments so all calculations and statistical analyses were carried out using means of the two tillage treatments for each main plot to make results more robust. Comparisons between treatments were carried out using ANOVA and Tukey as posttest (GraphPad Software, Inc., San Diego, CA).

## 3. Results

### 3.1. First Year

First year results refer to yields and grain characteristics of the three continuous wheat treatments. Plots which received sewage sludge at the beginning of the growing season showed the highest production of grain (*∼*6 t ha^−1^) and straw incorporation treatment the lowest (Table 2). However, mean values of “sldg” differed significantly from “strw” but not from the “cnvl” treatment. Sludge application resulted in grain N concentration (and total protein) similar with “cnvl”, but smaller 1000-kernel weight. Plots did not differ in number of spikes m^−2^, and grains did not differ in volume weight (Table 2). The amount of straw harvested from the “sldg” (7.8 t ha^−1^) was significantly greater in relation to “strw” but not to “cnvl.”

Grain and straw yield in combination with N concentrations in these two plant compartments gave estimates of the total amounts of N removed from soil after harvesting. These amounts were in the order “sldg” > “cnvl” > “strw” and were significantly different between treatments. Calculated N outputs (149.3, 104.1, and 177.6 kg ha^−1^ in “cnvl”, “strw,” and “sldg,” resp.) were in all cases higher than inputs through inorganic fertilization (91 kg ha^−1^ in “cnvl” and “strw” only). Total amounts of N in “cnvl” were 43% greater than in “strw.” This was due to greater overall production and grain N concentration but not to straw N concentration.

### 3.2. Second Year: Test Crop

Wheat was the test crop this year, and it was grown in all experimental plots.  Table 3 shows the results for this second year of the experiment (2007/08), which is the year of wheat cultivation after vetch or one year of fallow. As has already been mentioned, this cultivation period (November–May) was characterized by remarkably low rainfall. Wheat production, therefore, was from 50 up to 70% lower than the corresponding production of the previous year at the same plot. For “sldg,” second year results show residual effect of sewage sludge incorporation while results of “strw” and “cnvl” refer to a second consecutive year of straw application and conventional cultivation practices, respectively. Based on grain yields, treatments were ranked in the following order: “vinc” > “vhay” > “vgrn” > “falw” > “cnvl” > “sldg” > “strw” (Table 3). Vetch treatments performed better while “strw” was the worse for second consecutive year. Sludge maintained yield at levels equivalent to “cnvl.”

Volume weight of wheat grain was not significantly different among treatments, and 1000-kernel weight showed statistically significant lighter grains of “vinc” in relation to all other treatments apart from “cnvl” and “falw.”

**Figure 2:**
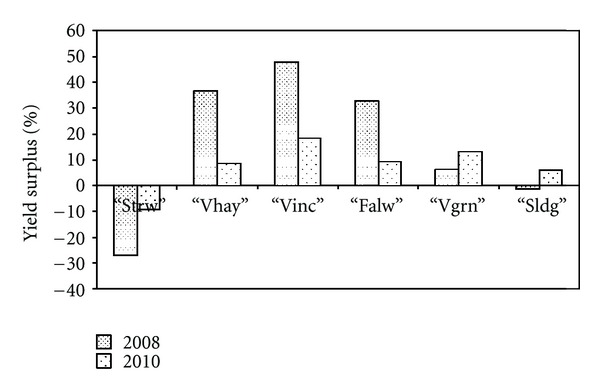
Percentage difference of grain yield of each treatment in relation to the conventional treatment at the very dry year of 2007/08 and the normal rainfall year of 2009/10.

 The effect of rotations or agricultural practices is better illustrated in Figure 2 showing the percentage yield surplus of the different treatments in relation to the conventional treatment. Vetch resulted in from 33 up to 48% higher yields of the subsequent wheat in relation to continuous wheat cultivation.

The comparison between treatments for straw production (Table 3) showed that the percentage difference in relation to conventionally cultivated plots was smaller than that for grain results. The exception was “falw,” which showed the same percentage increase in relation to conventional cultivation both for grain and straw production.

Differences in yield for grain or straw did not always correspond with the respective differences in the accumulation of nitrogen in plant tissues. Kjeldahl N in above-ground biomass (grain + straw harvested) per unit of area was significantly greater in the “vinc” plots and significantly smaller in “strw” plots in relation to the other treatments. Differences in total N removed from soil were mainly due to differences in the amount of N accumulated in grain as the amount of N found in straw was significantly different only for two pairs of treatments: “vinc”–“strw” and “vinc”–“sldg.”

In some treatments, biomass surplus or shortage in relation to “cnvl” biomass is accompanied by a respective change of total N accumulated in plant tissues. The ratio of N per unit of biomass (Table 3) was introduced as a means to express an eventual increase in concentration of N in plant tissues or “dilution” of N when biomass surplus does not correspond to greater N uptake. This ratio was greater than the respective one for the “cnvl” in the case of “vinc,” smaller in the case of “sldg,” and especially of “strw,” and almost the same for “vhay,” “vgrn,” and “falw.”

Grain harvest index (Table 3) showed clearly that a greater part of total production was “invested” to grain in the three vetch treatments contrary to “strw,” which showed the smallest index.

Results, especially of grain and straw production, tested the effectiveness of block separation as a means to control for experimental field variability. *P* value for differences between blocks was in the above cases much smaller than 0.05 justifying the initial experimental design of randomized blocks.

## 4. Discussion

Yields and N assimilation by test crop supported the well-documented hypothesis that cereals following N-fixing legumes in rotation systems have greater productivity [4, 13]. In the present study, the positive effect of one year's cultivation of common vetch on next year's crop yield in a very dry year was found to be greater than in a normal year at the same soil conditions (Figure 2) and greater than what has already been manifested in Cyprus [14, 15]. Long-term rotation experiments, with vetch followed by barley, have steadily demonstrated legume's positive effect, which, however, was never as great as in the current experiment even when barley was fertilized. The remarkable wheat yield surplus (47%) in this study was obtained at very limiting-rainfall conditions, which was expected to mask soil N availability effects [16], if the provision by legumes of biologically fixed N to next crop was the only mechanism by which these crops benefit subsequent cereals. Water stress has been shown in many occasions to reduce N uptake and consequently N fertilizer use efficiency [17, 18]. In the present experiment, results for biomass N per dry weight (Table 3) showed that greater plant growth of wheat after vetch incorporation was associated with increased uptake of soil N. Further research is, therefore, needed to reveal vetch effects in rotations, paying particular attention to cereal crop's nitrogen use efficiency and water use efficiency (e.g., [1]). These authors showed that vetch did not deplete soil moisture to the same extent as chickpea and medic, leaving some residual soil moisture for the succeeding wheat crop. They concluded that on the system basis, the wheat/lentil or wheat/vetch systems were most efficient at using rainfall. In addition, it has been shown [19] that water use efficiency increases with N fertilization, and therefore by increasing soil N availability, plants would be possible to withstand drought spells.

Hence, it is suggested that legume rotation yield surpluses is the result of the synergistic effect of water storage in soil and greater soil N availability and is probably the most important option for increasing the rainfall use efficiency of rainfed crops [20]. The inclusion of vetch in the rotation also resulted in the conversion of a greater part of the produced wheat biomass into grain, that is, greater harvest Index. Undoubtedly, nevertheless, rotation systems containing vetch should be characterized as systems possessing greater stability, that is, greater consistency of production [21]. This is a very important agroecosystem property under the fluctuating-rainfall conditions of dry areas, which has not been sufficiently emphasized until now. This property should be considered as an additional advantage to the already proven superiority of the vetch-cereal systems to be more sustainable, to minimize inputs, and to increase productivity and grain quality characteristics.

Conservation of soil water during fallow, which has been called upon as an explanation of increased yields after fallow [22], may explain the greater wheat yield found in this treatment. Moreover, other prerequisites for this positive effect to occur were operating such as a normal fallow-year rainfall and much lower rainfall at the wheat year and a deep heavy-textured clay soil for water storage [23]. It has to be noted, though, that under the climatic conditions of Cyprus, fallow did not always result in water storage in soil, and this water did not always have a positive effect to the subsequent cereal [7]. Fallow may also affect the rate of soil nutrient provision to subsequent crop, but, this is related to the rate of decomposition of the wild vegetation litter biomass appearing during fallow. From this point of view, apart from soil humidity, the number and timing of cultivations determining the amount and quality of biomass that is incorporated in soil should be considered as important controls of the fallow effects. Results on the overall concentration of N in plants of the present experiment, however, do not show any synergism between water use efficiency and N availability as far as the effect of fallow on wheat yield surplus is concerned.

Sludge effects during drought conditions are expected to be smaller than at normal rainfall. This is because a great part of its positive effect on wheat yield is attributed to the microbial release of nutrients from organic sources [24], which is highest at optimum soil moisture levels. It is emphasized that the 2007/08 drought occurred at the second year after application of sludge, when the provision of nutrients to plants relies more on the mineralization of organic forms of N and P. Despite that, this mineralization apparently provided sufficient amounts of nutrients to sustain a satisfactory yield without any other fertilizer addition.

The expected immobilization of N from the incorporation of plant residues with elevated C/N ratio [25, 26] is anticipated to be the main reason for the smaller grain and straw production and the inferior grain protein content in straw treatment. Any eventual positive influence on soil fertility arising from the incorporation of straw, such as the improvement of physical soil properties and water retention capacity or increase in soil organic matter, would probably require more time to become evident.

Productivity improvement measures in rainfed lands will have to be more extensively applied as there are physical, economic, and environmental limits in the exploitation of irrigation. Considerable alternatives are provided by fallowing or sewage sludge application under preconditions, but mainly by legume inclusion in rotation systems. This type of rotation appeared to be particularly effective in very dry years, which regularly occur in most Mediterranean countries.

## Figures and Tables

**Table 1:** Some physical and chemical properties of the soil in the experimental site and the incorporated sewage sludge.

	Total N (%)	NH_4_ (ppm)	NO_3_ (ppm)	pH	Organic matter (%)	Conductivity (mS/cm)	CaCO_3_ (%)	Sand (%)	Silt (%)	Clay (%)
Soil	0.085	6.2	5.0	7.94	0.93	0.143	15	39	26	35
Sludge	3.6%	1072.4	117.6	5.67	18.8	6.13				

**Table 2:** Mean values of grain and straw yields, grain yield components, and N concentrations in the plant parts harvested after the first year of experimentation. Values followed by the same letter do not show statistically significant differences between treatments at *P* < 0.05.

	“*Cnvl*”	“*Strw*”	“*Sldg*”
Grain production (t ha^−1^)	5.60 a	4.02 b	6.03 a
Volume weight (g l^−1^)	79.8 a	79.7 a	79.1 a
1000-kernel weight (g)	61.5 a	61 ab	58 b
Spike number (m^−2^)	213.9 a	198.3 a	232.9 a
Straw production (t ha^−1^)	6.7 a	5.5 b	7.8 a
N in grain (kg ha^−1^)	123.4 a	82.1 b	139.5 a
N in straw (kg ha^−1^)	25.9 a	22 a	38 b
Total N in plant (kg ha^−1^)	149.3 a	104.1 b	177.5 a

**Table 3:** Mean values of grain and straw yields, grain yield components, grain quality characteristics, and N concentrations in the plant parts harvested after the second year of experimentation. Values followed by the same letter do not show statistically significant differences between treatments at *P* < 0.05.

	*“Cnvl”*	*“Strw”*	*“Vhay”*	*“Vinc”*	*“Vgrn”*	*“Falw”*	*“Sldg”*
Grain production (t ha^−1^)	2.19 cd	1.60 d	2.99 ab	3.24 a	2.91 ab	2.33 bc	2.16 cd
Volume weight (g l^−1^)	160.02 a	160.28 a	162.9 a	161.1 a	162.26 a	160.6 a	160.28 a
1000-kernel weight (g)	50.56 ab	53.81 a	51.69 a	47.69 b	52.06 a	50.44 ab	52.19 a
Spike number (m^−2^)	56.6 a	49.8 a	54.9 a	60.6 a	51.6 a	58.9 a	49.5 a
Straw production (t ha^−1^)	3.88 ab	3.42 b	3.94 ab	4.67 a	4.12 ab	4.13 ab	3.54 b
N in grain (kg ha^−1^)	46.44 bc	27.69 c	60.26 ab	78.71 a	61.21 ab	46.71 bc	42.44 bc
N in straw (kg ha^−1^)	19.55 ab	11.72 b	15.97 ab	23.21 a	17.13 ab	18.84 ab	12.63 b
Total N in plant (kg ha^−1^)	65.99 b	39.42 c	76.24 b	101.92 a	78.35 ab	65.54 b	55.07 bc
Grain harvest index	0.355 cd	0.316 d	0.428 a	0.406 abc	0.408 ab	0.359 bcd	0.375 bc
N in biomass (%)	1,08 ab	0,79 c	1,09 ab	1,30 a	1,10 ab	1,02 bc	0,95 bc
